# Impulsivity and Concussion in Juvenile Rats: Examining Molecular and Structural Aspects of the Frontostriatal Pathway

**DOI:** 10.1371/journal.pone.0139842

**Published:** 2015-10-08

**Authors:** Harleen Hehar, Keith Yeates, Bryan Kolb, Michael J. Esser, Richelle Mychasiuk

**Affiliations:** 1 Alberta Children’s Hospital Research Institute, University of Calgary, Faculty of Medicine, Calgary, Canada; 2 Alberta Children’s Hospital Research Institute, University of Calgary, Department of Psychology, Calgary, Canada; 3 Canadian Centre for Behavioural Neuroscience, University of Lethbridge, Lethbridge, Canada; University of Colorado, UNITED STATES

## Abstract

Impulsivity and poor executive control have been implicated in the pathogenesis of many developmental and neuropsychiatric disorders. Similarly, concussions/mild traumatic brain injuries (mTBI) have been associated with increased risk for neuropsychiatric disorders and the development of impulsivity and inattention. Researchers and epidemiologists have therefore considered whether or not concussions induce symptoms of attention-deficit/hyperactivity disorder (ADHD), or merely unmask impulsive tendencies that were already present. The purpose of this study was to determine if a single concussion in adolescence could induce ADHD-like impulsivity and impaired response inhibition, and subsequently determine if inherent impulsivity prior to a pediatric mTBI would exacerbate post-concussion symptomology with a specific emphasis on impulsive and inattentive behaviours. As these behaviours are believed to be associated with the frontostriatal circuit involving the nucleus accumbens (NAc) and the prefrontal cortex (PFC), the expression patterns of 8 genes (*Comt*, *Drd2*, *Drd3*, *Drd4*, *Maoa*, *Sert*, *Tph1*, *and Tph2*) from these two regions were examined. In addition, Golgi-Cox staining of medium spiny neurons in the NAc provided a neuroanatomical examination of mTBI-induced structural changes. The study found that a single early brain injury could induce impulsivity and impairments in response inhibition that were more pronounced in males. Interestingly, when animals with inherent impulsivity experienced mTBI, injury-related deficits were exacerbated in female animals. The single concussion increased dendritic branching, but reduced synaptic density in the NAc, and these changes were likely associated with the increase in impulsivity. Finally, mTBI-induced impulsivity was associated with modifications to gene expression that differed dramatically from the gene expression pattern associated with inherent impulsivity, despite very similar behavioural phenotypes. Our findings suggest the need to tailor treatment strategies for mTBI in light of an individual’s premorbid characteristics, given significant differences in molecular profiles of the frontostriatal circuits that depend upon sex and the etiology of the behavioural phenotype.

## Introduction

Many developmental disorders, such as attention-deficit/hyperactivity disorder (ADHD) are believed to have multifactorial etiologies involving alterations to several neural pathways [1]. These variations in pathogenesis contribute to substantial heterogeneity in symptom presentation and diagnosis [2, 3]. In essence however, ADHD is a disorder of executive function that is characterized by a pervasive pattern of inattention, impulsivity, and hyperactivity that are developmentally inappropriate [4]. Although all symptoms are potentially debilitating, impulsivity is now recognized as having a central role in the pathogenesis of many neuropsychiatric disorders [5]. In the broadest of terms, impulsivity is characterized by poor self-control and reflected in rapid decision-making that lacks foresight and anticipation of future consequences. Two main pathways of executive function, response inhibition and reward signaling, seem to be critical for impulse control and the ability to delay gratification. Alterations to these pathways, which involve the prefrontal cortex and dorsal striatum, are hypothesized to result in dysfunctional processing of anticipated rewards and a reduction in cognitive flexibility [6]. Research has articulated complex and interdependent roles for dopamine and serotonin systems in the etiology of impulsivity. These roles have been corroborated by efficacious drugs that act upon dopamine receptors in the nucleus accumbens (NAc) (for review see [5]). In addition, extensive connections exist between serotonergic neurons and many of the brain regions involved in the regulation of impulse control, such as the NAc, prefrontal cortex (PFC), ventral tegmental area (VTA), and hippocampus [7, 8].

Owing to the strong hereditary influence on ADHD prevalence (71% of variance accounted for by genetic differences), many researchers have attempted whole genome linkage studies and genome wide association studies (GWAS) (for review see [1]). Unsurprisingly, strong correlations were consistently found for many dopaminergic and serotonergic genes, but a single common genetic variant is unlikely to be responsible for individual presentations of impulsivity. Researchers have therefore begun to postulate that cell architecture and function may play a more significant role than originally believed [1]. In support of this, resting-state functional connectivity MRI (rs-fcMRI) studies have found positive linear relationships between NAc—PFC connectivity and impulsivity [6]. Moreover, a meta-analysis of 55 different studies demonstrated that the executive dysfunction associated with impulsivity and inattention resulted from *hypoactivation* of the frontoparietal network, whereas distractibility was linked to *hyperactivation* of the ventral attention network [9]. To our knowledge no studies have looked at dendritic morphology with respect to impulsivity, but neurotransmitter modulation has been investigated extensively (for review see [5]). For example, mice lacking the dopamine transporter 1 (*Dat1*) exhibit hyperactivity and risk taking behaviours that reflect impulsivity [10], while specific human *Dat1* genotypes have been linked to striatal activation patterns in ADHD individuals [11]. A novel study by Dalley and colleagues [12] showed that impulsivity in rats was associated with a significant reduction in dopamine 2 and dopamine 3 receptors in the ventral striatum.

A major contributor to the occurrence of impulsivity, and possibly ADHD in adolescence is mild traumatic brain injury (mTBI) or concussion [13, 14]. TBI, generally associated with falls, sports, and automobile accidents, is the leading cause of disability and mortality in children and adolescents [15], with mTBI and concussions accounting for 80–90% of all of these injuries [16]. Although symptoms are transient for most children, a significant proportion of children go on to suffer from lingering and progressive symptoms [17]. Studies indicate that children with persistent post-concussive symptoms experience difficulties paying attention, decreased concentration, and slowed reaction times, while their caregivers often report increased impulsivity and impatience [18–21]. As concussion symptomology is likely dependent upon reductions in tract integrity associated with tearing and shearing of white matter [22–25], it stands to reason that the complex circuits involved in reward processing and response inhibition are also likely to be at risk. Circumstantial differences in directionality and severity of the acceleration forces involved in the brain injury, in conjunction with pre-morbid characteristics, could therefore contribute to the heterogeneity of symptom presentation [7, 25–27].

With this in mind, the purpose of the current study was two fold. First, the study sought to examine the effects of an adolescent mTBI on aspects of the reward pathway, with specific emphasis on the frontostriatal circuit. Investigation into this pathway involved implementation of multiple techniques, including behavioural analysis of impulsivity and response inhibition, Golgi-Cox staining of medium spiny neurons in the NAc, and determination of gene expression changes in both the PFC and NAc. The second objective of the study was to determine how pre-existing impulsivity moderated outcomes from an early mTBI, with an emphasis on the frontostriatal reward circuit. Two behavioural paradigms capitalized on Go/No-Go training to examine impulsivity, response inhibition, and strategy perseveration, while gene expression changes in dopamine receptors, catecholamine transporters, and neurotransmitter signaling were used to study aspects of the frontostriatal circuit in impulsive and standard rats after a concussion. Based upon epidemiological data [28], we hypothesized that the adolescent brain injury would disrupt normal patterning of the frontostriatal pathway, and that premorbid impulsive behaviours would exacerbate mTBI-induced deficits of attention and inhibition

## Materials and Methods

### Study Design

The study was divided into two distinct but related experiments. As both experiments used the same protocols and procedures, they will only be described once. The first experiment was designed to examine the effects of a concussion on the frontostriatal pathway in standard juvenile rats. Male and female pups were trained on the paradigms below, received an early brain injury, underwent testing, and were sacrificed for molecular and neuroanatomical analysis. The second experiment was designed to investigate the effects of pre-morbid impulsivity on mTBI-related modification to the frontostriatal pathway. Male and female pups from the standard and impulsive cohorts were trained on the paradigms below, received an early brain injury, underwent testing, and were sacrificed for molecular analysis.

### Subjects and Breeding Procedures

All experiments reported here were carried out in accordance with the Canadian Council of Animal Care and received approval from the University of Calgary Conjoint Faculties Research Ethics Approval Board. The husbandry room was maintained at 21°C on a 12:12 hr light:dark cycle in which the lights turned on at 0700. Half of the Sprague Dawley rat pups were born to dams that had *ad libitum* access to food and water (STD) and the other half were born to dams that were calorically restricted for 9 weeks; 3 weeks prior to mating, the 3 weeks of gestation and the 3 weeks of weaning (IMP). In the caloric restriction group, dams had *ad libitum* access to food every other day; food was restricted on alternating days but access to water was maintained throughout. Care was taken to ensure that dams did not lose more than 30% of their average daily weight. As prior observations in the laboratory [29] suggested that pups born to calorically restricted dams were hyperactive and possibly impulsive, we used this programming technique to generate an inherently impulsive phenotype that could be tested in this study. A total of 41 pups were born to four calorically restricted dams (23 Male: 18 Female) and 42 pups were born to four standard fed dams (20 Male: 22 Female). To control for litter effects, at postnatal day 21 (P21) a single male and female from each dam was randomly assigned to live in a sex-matched cage with 3 other animals from the same dam type (STD vs. IMP). A total of 48 rat pups (12 male STD, 12 male IMP: 12 female STD, 12 female IMP) were used for this study.

### Hyperactivity

Animals were tested in the Open Field paradigm on P26, P33, P40, P47, and P55 to measure general locomotor activity. On each of these days, rats were placed in the center of a circular arena (diameter 135 cm) and permitted to explore the environment for 10 minutes. An overhead camera running Noldus Ethovision XT 10.0 software was used to track and analyze the rat’s overall movement and distance travelled. The arena was cleaned with Virkon® between each testing session.

### 5-Choice Serial Reaction Test (Go/No-Go Task & Extinction Paradigm)

All animals began training in the 5-Choice Serial Reaction test (Go/No-Go task) on P27. For the duration of Go/No-Go training and testing, rats were food restricted in an effort to increase their motivation to obtain the food rewards associated with the task (weight loss did not exceed 20% of normal body weight). The training protocol used was similar to that of Bari et al [30], but modified by this laboratory [31] for younger rats. The protocol ran on two identical Habitest Modular 5-Hole Operant Conditioning Chambers (28 cm x 29 cm x 24 cm–W x H x D) (Harvard Apparatus, QC Canada). The training was divided into two stages. The first step was designed to teach the rats that a reward (a banana flavored 45g precision-weight food tablet (BioServ, Product #F0059)) would be provided when they correctly nose-poked an illuminated hole (Go stimuli) and that 3 reward pellets would be provided if they resisted nose-poking an illuminated hole when all of the holes were illuminated (No-Go stimuli). In summary, Stage 1 training sessions were 20 minutes long. They began with an illumination of the house light and reward magazine where a reward pellet had been left. Once the rat retrieved the reward pellet, the session commenced with the house light and reward magazine light being extinguished and the Go stimulus initiated. The Go stimulus in this stage consisted of illumination of 1of the 5 holes; this hole remained illuminated until the rat nose-poked the specific hole. Upon nose-poking the correct hole, the hole light extinguished and the reward magazine/house lights illuminated at the same time that a banana pellet was dispensed into the reward magazine. During Stage 1 of the training, the rat was not penalized for nose-poking the wrong hole; it was simply just not rewarded, i.e. nose poking of the other 4 dark holes would not dispense a banana pellet nor would it extinguish the Go stimulus. Once the rat correctly nose-poked and retrieved its banana pellet, a second hole was illuminated (the order of the Go stimuli illumination was randomly designed) and the process continued. No-Go stimuli were randomly presented (~ every 5–7 stimuli), interspersed among the Go stimuli. A No-Go stimulus was characterized by simultaneous illumination of all 5 holes. The rat had to learn to abstain from nose poking under these conditions. If the rat was able to abstain from responding in the No-Go stimulus, the lights in the 5 holes were extinguished, the reward magazine and house lights were illuminated, and 3 banana pellets were dispensed. An incorrect response on the No-Go stimuli (nose-poking any of the illuminated holes) resulted in a 20 second time-out (a period of darkness where the rats were unable to poke for rewards). Stage 1 training sessions occurred once/day for 10–14 consecutive days. When rats were proficient at Stage 1, Go stimuli, animals were switched to Stage 2 training.

Stage 2 of the training procedure required sustained visual attention. Similar to Stage 1, Stage 2 began with illumination of the house light and reward magazine and retrieval of a banana pellet. However, when these lights were extinguished a Go stimulus was again initiated (illumination of a single hole), but it only remained illuminated for 5 seconds. The animal was now required to nose poke within the 5s illumination period to receive a reward. If the animal nose-poked within the limited period, the illumination of the hole was extinguished, the house light and reward magazine were illuminated, and the banana pellet dispensed. Conversely, if the rat failed to nose poke the correct hole in the 5 s illumination period, it received a 5 s timeout. Once the rat retrieved the reward or the time-out ended, a second Go stimulus was illuminated (again the Go stimuli were delivered in a random order that differed from Stage 1). No-Go stimuli were also randomly injected into the Stage 2 training session, approximately every 5–7 stimuli. The No-Go stimuli procedure did not differ from that described in Stage 1. Stage 2 sessions were also 20 minutes long and occurred over 21 consecutive days.

Stage 3 was an Extinction protocol that was delivered over 3 consecutive days. This procedure was designed to determine if the animals would continue to nose poke in the absence of a reward. The rats were again placed in the Habitest Modular 5-Hole Operant Conditioning chambers for 20-minute sessions. The Extinction procedure consisted of only Go stimuli (5 s illuminations of a single hole). However in these circumstances when the rat correctly nose-poked, no banana pellet rewards were dispensed, i.e. the illumination would be extinguished, but there would be no subsequent illumination of the reward magazine and house light or deliverance of any pellets. After a 5 s delay, another Go stimuli would be illuminated. If correctly nose-poked in the 5 s period, the light would again extinguish but not be followed by a reward. If the rat did not correctly nose poke in the 5 s period, the light would extinguish, there would be a 5 s delay and another light would illuminate. There were no No-Go stimuli in the Extinction stage.

All data for the Go/No-Go test and Extinction paradigm was collected and analyzed with Graphic State 4 software (Coulburn Instruments, QC Canada). Data was collected for the Go/No-Go test at two distinct time-points, the day prior to the mTBI (7 days of Stage 2 training had been completed) and on the last day of Stage 2 training. Data for the Extinction paradigm was collected on Day 1 and Day 3 of Extinction training. Rats were scored on 5 measures; A) their ***accuracy*** level determined by their ability to detect the illumination and respond correctly by nose-poking the appropriate hole, B) their level of ***inaccuracy*** determined by nose-poking the wrong hole during the 5 s illumination period; given that illumination remained for 5 s or until the rat correctly nose-poked, rats could incorrectly nose poke multiple wrong holes for any given Go stimulus, C) ***impulsivity***—additional nose-pokes into any of the holes that occurred during a time-out or premature nose-pokes that occurred in the 5 s delay after reward retrieval and before the next light could be illuminated, D) ***response inhibition—***the latency to nose poke on the No-Go stimuli, and E) the number of ***missed lights*** which measured the number of lights that were illuminated for 5 s but not correctly nose-poked.

### mTBI Procedure and Validation

At P48, rats from both of the cohorts (IMP and STD) received a mTBI using the modified weight drop technique as previously described [32, 33] or a sham injury. In short, rats were lightly anesthetized (until non-responsive to a toe pinch) with isofluorane and placed chest down on a scored piece of tinfoil that was suspended 10cm above a foam collection sponge. A 150g weight was dropped through a Plexiglas® guide tube (from 0.5m height), which produced a glancing impact to the top of the rat’s head propelling it through the tinfoil. The rat underwent a 180° rotation and landed on its back. The free fall produced acceleration/deceleration and rotational forces on the brain that mimic the forces of concussion. Immediately after the injury, the rats received topical administration of lidocaine and were placed in the supine position in a clean warm cage to recover. Animals experiencing a sham injury were exposed to the same procedure but did not experience the glancing impact or free fall.

**Fig 1:**
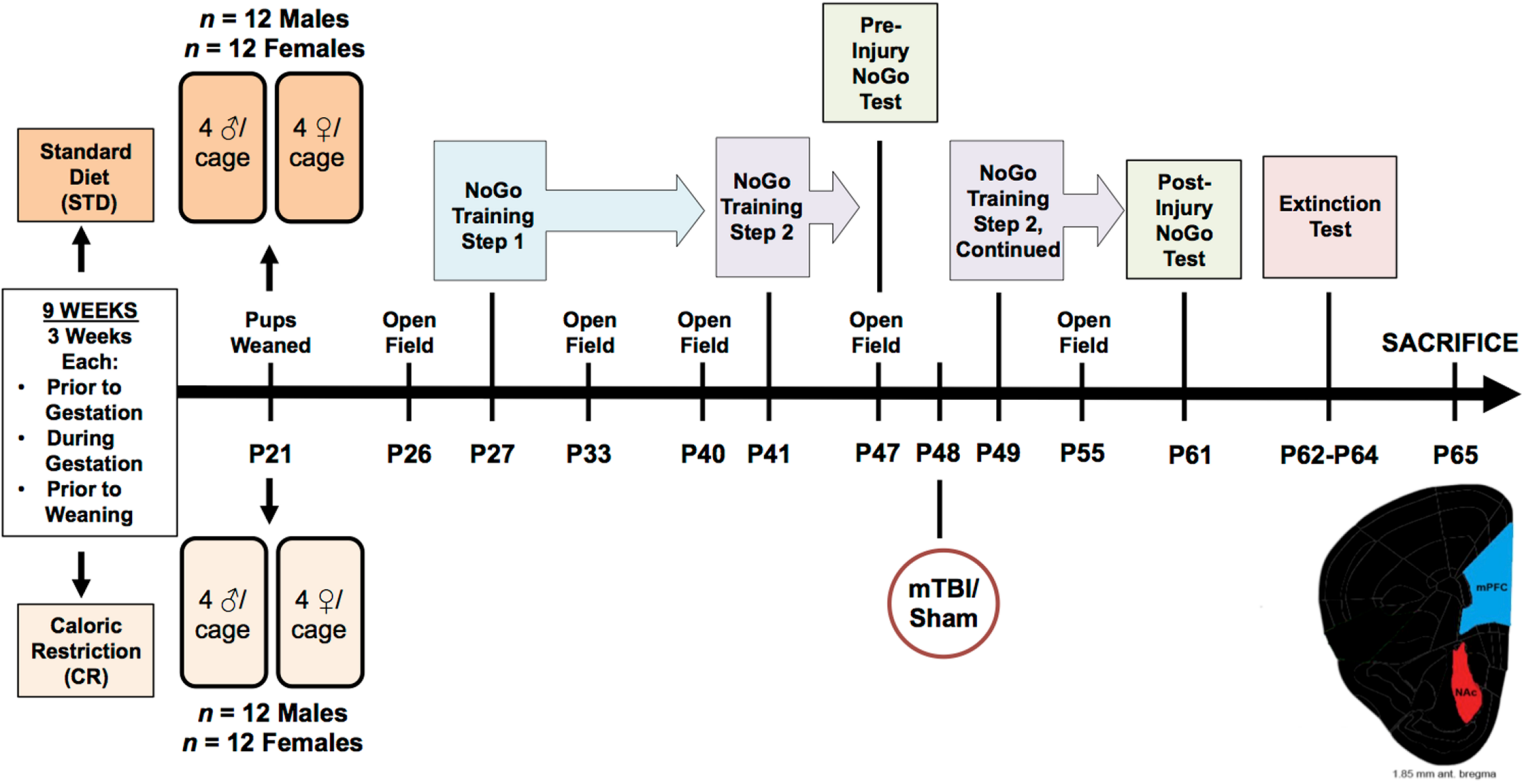
Illustrative representation of the experimental paradigm, which includes the age that each rat underwent behavioural testing, mTBI, and sacrifice.

Following the injury, all animals were scored for their **time-to-right**. This was measured as the time each rat took to wake and right its self, i.e. flip from the supine position to a prone or standing position. This was used a measure of loss of consciousness that differentiates between average time to wake from just the anesthetic (control animals) and time to wake after the anesthetic plus the injury (mTBI animals). Animals that had experienced a mTBI took significantly longer to wake and right themselves than animals in the sham group, regardless of sex or cohort, *F*(1, 41) = 26.19, *p* < .01. [Male IMP mTBI; 33.31 ±2.6 sec, Male IMP sham; 16.67 ±2.6 sec, Male STD mTBI, 26.82 ± 3.0 sec, Male STD sham; 18.32 ± 3.0 sec, Female IMP mTBI; 31.27 ±2.6 sec, Female IMP sham; 22.9 ±2.6 sec, Female STD mTBI; 34.71 ±3.0 sec, Female STD sham; 19.46 ±3.0 sec]. This finding is consistent with prior studies in our laboratory and is used to validate the presence of a brain injury [32, 34]. See Fig 1 for an overview of the experimental design along with a timeline of important testing dates.

### Neuroanatomical and Molecular Analysis

Following all behavioural testing, rats were sacrificed at P65. A portion of the rats (*n =* 28) were subjected to isoflourane inhalation, weighed, and rapidly decapitated. Using the Zilles atlas [35] tissue from the Cg3 and IL (the PFC) and NAc was removed, flash frozen on dry ice, and stored at -80°C for molecular profiling. The remainder of the rats (*n* = 20) were administered an overdose of sodium pentobarbital, weighed, and intracardially perfused with 0.9% saline. The brains were removed and placed in dark bottles containing Golgi-Cox solution where they were stored for 14 days.

For the molecular analysis, total RNA was extracted from the brain tissue with the Allprep RNA/DNA Mini Kit according to manufacturer protocols (Qiagen, Germany). The concentration and purity of samples were measured with a NanoDrop 2000 (Thermo Fisher Scientific, USA). Purified RNA (2μg) was reverse transcribed into cDNA using the oligo(dT)_20_ Superscript III First-Strand Synthesis Supermix Kit (Invitrogen, USA) according to manufacturer protocols.

Genes were selected based on their prior use in studies of impulsivity, with specific emphasis on the frontostriatal pathways. Eight genes were selected: Catachol-O-methyltransferase (*Comt*), Dopamine receptors 2, 3, and 4, (*Drd2*, *Drd3*, *Drd4*), Monoamine oxidase A, (*Maoa*), Serotonin transporter (*Sert*), and Tryptophan hydroxylase 1, and 2 (*Tph1*, *Tph2*). Multiple attempts were made to examine the Dopamine transporter (*Dat1*), but expression levels were below detectable ranges. In addition, *Drd3* expression was determined for the NAc, but fell below detection in the PFC and was therefore omitted for that specific brain region.

All primers for the qRT-PCR were designed in-house by a research technician using Primer3 (http://bioinfo.ut.ee/primer3) and were then purchased from IDT (Coralville, USA). See Table 1 for primer sequences and optimal cycling parameters for the 8 genes. Each sample was run in duplicate and two separate research analysts processed all target genes. qRT-PCR was performed and analyzed with the CFX Connect Real-Time PCR detection system (Bio-Rad, Hercules, USA) with 10ng of cDNA, 0.5μM of the forward and reverse primers, and 1X SYBR Green FastMix with Rox. Relative target gene expression was determined by normalization to two housekeeping genes, CycA and Ywhaz [36] using the 2^-ΔΔCt^ method as previously described by Pfaffl [37].

**Table 1:** Primer information for the 8 genes examined with relative qPCR.

Gene Symbol	Gene Name	Primer Sequence	Amplicon Size (bp)	Tm (°C)	Cycling Parameters
		(+ sense and–antisense)			
*Comt*	Catechol o-methyltransferase	(+) atcttcacggggtttcagtg	145	60.0	
		(-) gagctgctggggacagtaag			
*Drd2*	Dopamine receptor D2	(+) gccgagttactgtcatgattgc	154	60.0	
		(-) ggcacgtagaatgagacaatgg			
*Drd3*	Dopamine receptor D3	(+) tcttcagtggtgtccttctacg	200	59.0	
		(-) ctggcccttattgaaaactgcc			
*Drd4*	Dopamine receptor D4	(+) cctcaaccccatcatctacacc	193	60.0	1 cycle 95°C 3 min,
		(-) cccagcgttgataaatggttagg			
*Maoa*	Monoamine oxidase A	(+) gagaagaactggtgtgaggagc	170	60.0	40 cycles 95°C 15 sec,
		(-) tcaactgctccttccatgtagc			
*SERT*	Serotonin transporter (*Slc6a4*)	(+) gtggactctcagacatgctacc	190	60.0	40 cycles 95°C 15 sec,
		(-) ttagaagctaacagatgcgggg			
*Tph1*	Tryptophan hydroxylase 1	(+) cactcactgtctctgaaaacgc	216	60.0	40 cycles Tm °C 30 sec,
		(-) agccatgaatttgagagggagg			
*Tph2*	Tryptophan hydroxylase 2	(+) gtgtgtaaaagcctttgacccg	181	60.0	+Melt Curve
		(-) ttcaatgctctgtgtgtagggg			
*CycA*	Cyclophilin A	(+) agcactggggagaaaggatt	248	58.0	
		(-) agccactcagtcttggcagt			
*Ywhaz*	Tyrosine 3-monooxygenase/ tryptophan, 5-monooxygenase activation protein, zeta	(+) ttgagcagaagacggaaggt	136	56.1	
		(-) gaagcattggggatcaagaa			

**Fig 2:**
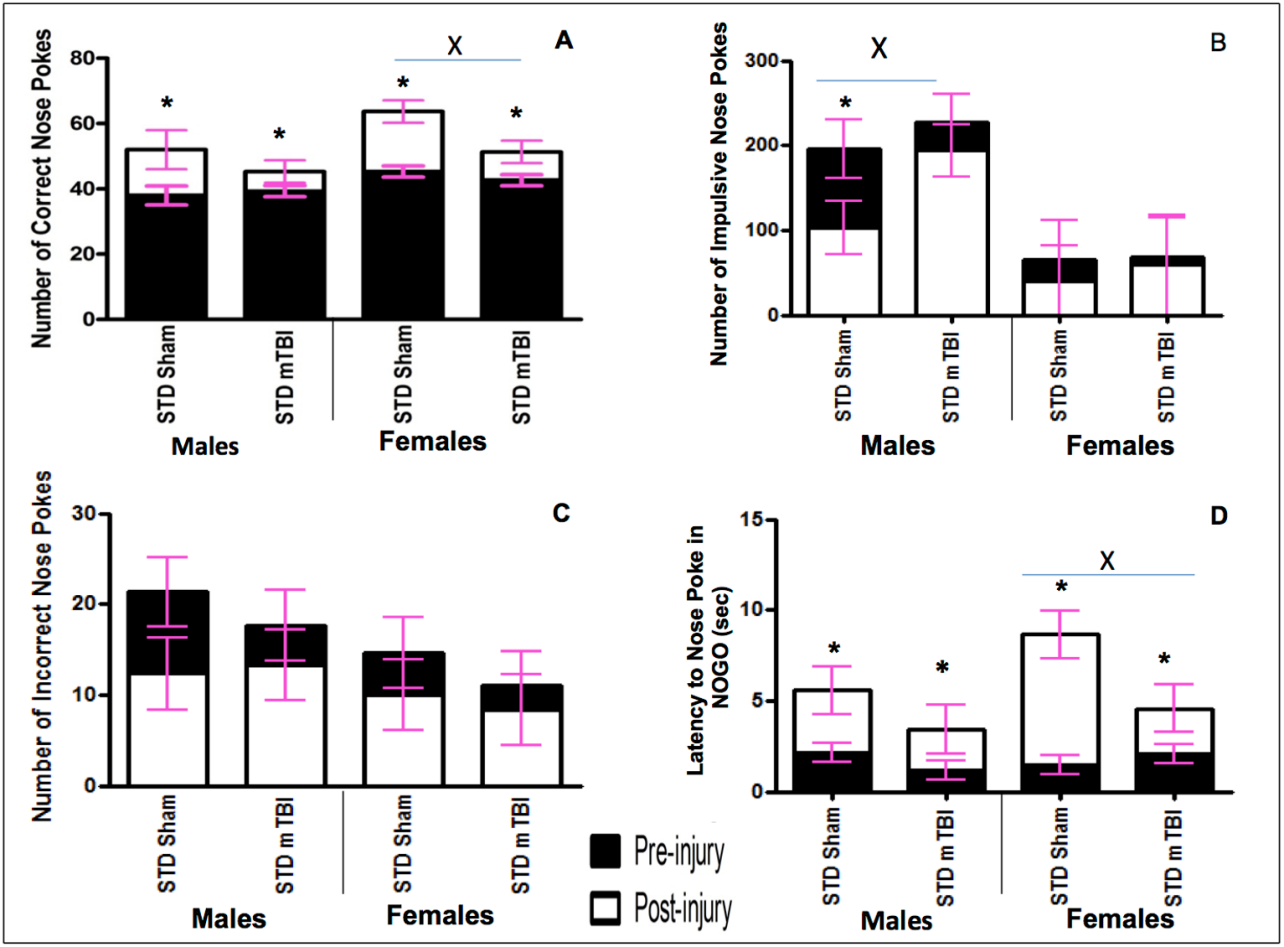
Graphical representation of Go/No-Go results. A) Accuracy, B) Impulsivity, C) Inaccuracy, and D) Response Inhibition, for male and female animals from the STD cohorts at the pre-injury (black bars) and post-injury (white bars) test sessions (x significant effect of mTBI, * significant effect of testing period, all *p* < .05).

To complete the histological processing, brains were transferred to 30% sucrose solution, following the 14-day Golgi-Cox impregnation period. Brains remained in sucrose for at least 3 day prior to being cut at 200μm on a Vibratome. Brain slices were mounted on gelatin coated slides and then stained according to the Golgi-Cox procedure described elsewhere [38, 39]. Individual neurons were selected from the NAc and traced at 250x using a camera lucida mounted on a microscope. A total of 10 cells (5/hemisphere) were traced from the NAc of each animal. The mean of cells from each hemisphere comprised the data points for the statistical analyses. See Fig 2B for a representative drawing of the cells and spines from the NAc. Neuroanatomical analyses included: 1) dendritic branch order, an overall estimation of the dendritic complexity based on the number of times the branches bifurcate, 2) Sholl analysis, a measure of dendritic length derived from the number of dendritic branches that intersect concentric circles spaced 20μm from the cell body, and 3) spine density, a measure of synaptic prevalence that is calculated by increasing the microscope to a 1000x magnification and counting the number of spine protrusions for every 10μm. A research technician blinded to all experimental conditions drew the neurons under investigation, and another research technician completed the analysis.

### Statistical Analysis

All statistical analyses were carried out with SPSS 22.0 for Mac. Repeated measures ANOVAs were conducted with the pre-injury and post-injury testing points (days) treated as within-subjects variables and sex (male/female), injury (mTBI/sham), and cohort (IMP/STD) treated as between-subject factors. Dependent variables were drawn from the Go/No-Go test. Repeated Measures ANOVAs were also conducted with extinction day 1 and extinction day 3 (days) treated as within-subjects variables and sex (male/female), injury (mTBI/sham), and cohort (IMP/STD) treated as between-subject factors with outcomes of the Extinction paradigm treated as dependent variables. A repeated measures ANOVA for the 5 testing days with sex (male/female), injury (mTBI/sham), and cohort (IMP/STD) as factors was run to analyze hyperactivity in the open field. Three-way ANOVAs with sex (male/female), injury (mTBI/sham), and cohort (IMP/STD) as factors were run for the measures of injury validation and changes in gene expression. Finally, a two-way ANOVA with sex (male/female) and injury (mTBI/sham) as factors was run for the neuroanatomical variables. In all cases a *p* < .05 was considered statistically significant.

## Results

### Experiment #1. Pediatric mTBI and the Frontostriatal Pathway

#### Go/No-Go Paradigm

Accuracy: Accuracy was used to measure the animal’s spatial attention and ability to quickly and accurately respond to the illumination of a random hole. Animals were tested in the Go/No-Go paradigm prior to the injury and approximately 2 weeks after the mTBI. All animals improved from the first testing period to the second, but the improvement in accuracy was significantly greater for sham animals than for mTBI animals. The repeated measures ANOVA demonstrated a significant day x injury interaction, *F*(1,19) = 9.12, *p* = .01, with a significant between-subjects effect of injury, *F* (1, 19) = 9.47, *p* = .01, sex, *F*(1, 19) = 7.61, *p* = .02, but not a significant interaction, *p* >.05. See Fig 2A.

Impulsivity: The number of ‘impulsive’ nose-pokes (nose-poking prior to activation of the Go stimulus, or during a timeout) was measured for both testing sessions. A mTBI induced a significant increase in the number of impulsive nose-pokes committed by male animals at the second testing session. No differences were observed in the number of impulsive nose-pokes for females. The repeated measures ANOVA demonstrated a significant between-subjects effect of sex, *F* (1, 19) = 45.53, *p* < .01, but not of injury, *F*(1, 19) = 2.13, *p* = .18. See Fig 2B.

Inaccuracy: Inaccuracy was a measure of the number of holes that were poked incorrectly while the cued light was on. There were no significant differences between sham and mTBI animals at the second testing session. The repeated measures ANOVA demonstrated no significant effects or interactions, *p’s* >.05. See Fig 2C.

Response Inhibition: Animals were scored on their ability to inhibit a response during the No-Go stimuli. Early in the training procedure (test day 1) all animals scored poorly on this task, regardless of sex. Similar to the accuracy testing, sham animals exhibited greater improvements from the pre-injury test to the post injury session than mTBI animals. The repeated measures ANOVA demonstrated a significant day effect, *F*(1, 19) = 26.41, *p* < .01, and a day x injury interaction, *F*(1,19) = 4.94, *p* = .05. See Fig 2D.

#### Neuroanatomical Analysis

Golgi-Cox analysis of neurons in the NAc was only carried out for animals in the standard cohort with a mTBI or sham injury. The analysis did not include IMP animals.

**Fig 3:**
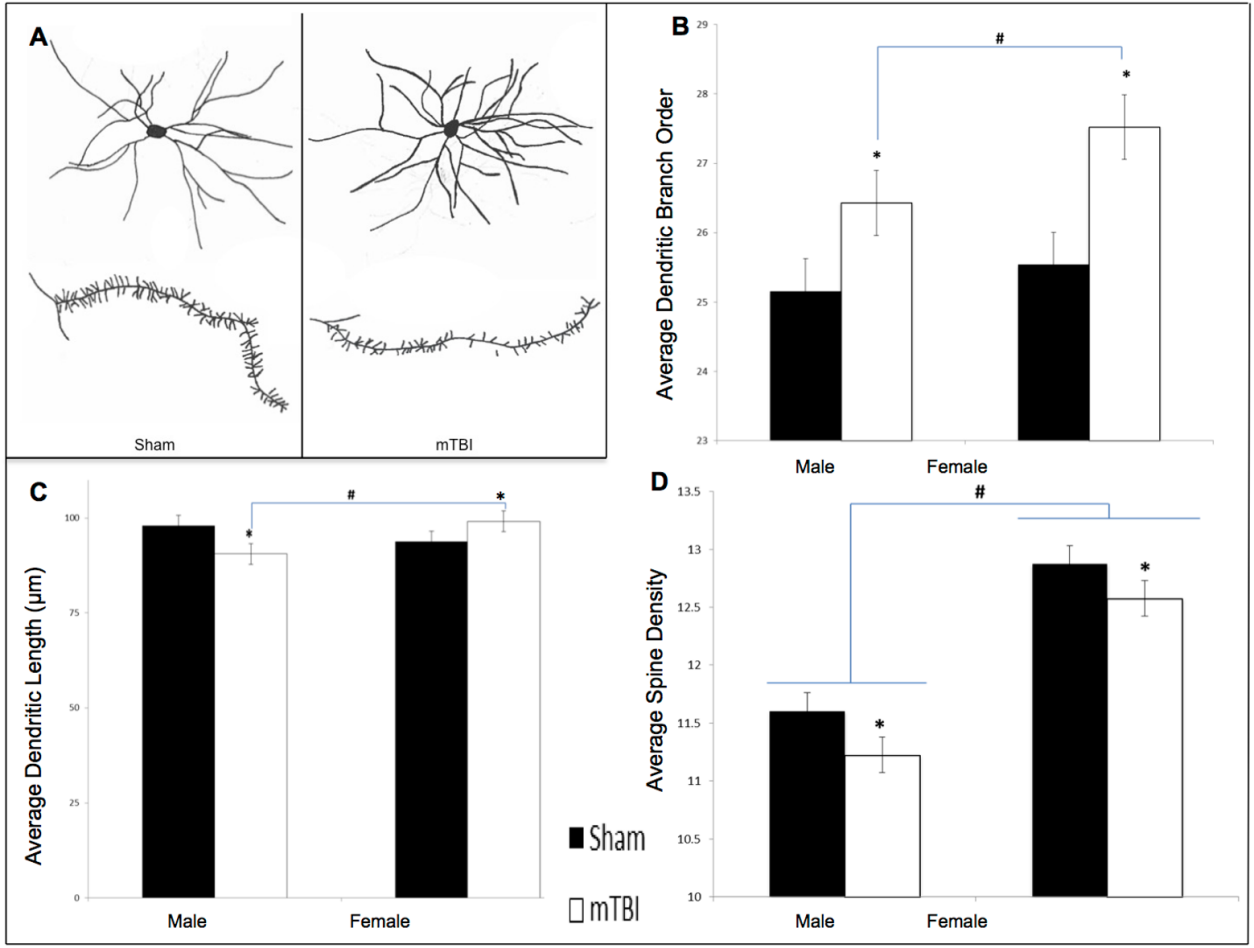
Neuroanatomical findings from STD animals for the NAc medium spiny neurons. A) exhibits a representative tracing of average medium spiny neurons and dendritic spines from the NAc in sham and mTBI animals; B) graphical representation of dendritic branch order from Golgi-Cox stained neurons; C) representation of average dendritic length, and D) is illustrative analysis of dendritic spine density; (*main effect of mTBI *p* < .05; # main effect of sex *p* < .05).

Dendritic Branch Order: Analysis of the medium spiny neurons in the NAc demonstrated that both male and female animals with an early mTBI had significantly more dendritic branches when compared to animals with a sham injury. The two-way ANOVA showed a main effect of injury, *F*(1, 39) = 12.09, *p* < .01, but not of sex, *F*(1, 39) = 2.54, *p* = .12, nor a significant interaction, *F*(1, 39) = 0.60, *p* = .45. See Fig 3B.

Sholl Analysis / Dendritic Length: The injury-dependent changes in dendritic length of medium spiny neurons varied for males and females. Male animals with an mTBI experienced significant reductions in dendritic length whereas female animals with an mTBI exhibited significant increases in dendritic length when compared to controls. The two-way ANOVA demonstrated a significant sex by injury interaction, *F*(1, 39) = 5.47, *p* = .03, but no significant main effects, *p*’s > .05. See Fig 3C.

Spine Density: Examination of synapse density in the NAc found that males and females differed at baseline, but both experienced a significant reduction following the mTBI. The two-way ANOVA demonstrated a significant main effect of sex, *F*(1, 39) = 73.27, *p* < .01, and of injury, *F*(1, 39) = 4.97, *p =* .03, but the interaction was not significant, *F*(1, 39) = 0.07, *p* = .80. See Fig 3D.

#### Molecular Analysis

**Fig 4:**
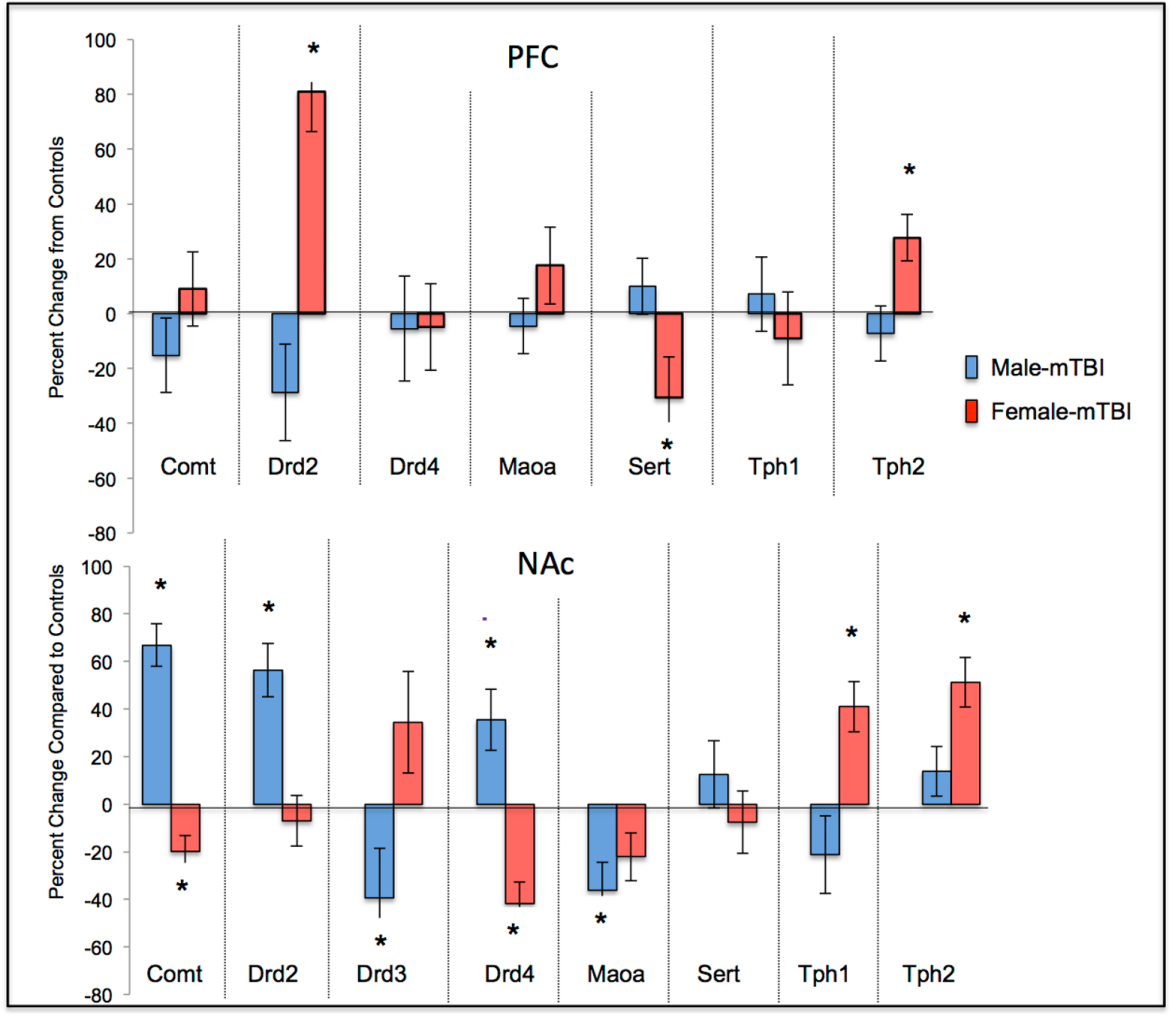
Changes in gene expression for STD male and female animals with a mTBI. The centerline represents typical expression (STD-shams) with the bars exhibiting percent changes from normal (*main effect of mTBI *p* < .05).

Results from the ANOVAs for the 8 genes examined can be found in Table 2. In summary, a majority of the genes examined in the PFC and NAc exhibited significant sex-differences or sex x mTBI interactions whereby the mTBI resulted in opposing changes in gene expression for females and males. See Fig 4.

**Table 2:** Summary of results from the two-way ANOVAs for changes in expression of each of the genes examined in the NAc and PFC of animals with a sham or mTBI F(p).

Brain Region	Gene	Sex Effect	mTBI Effect	Sex x mTBI Interaction
	***Comt***	3.05 (.12)	1.90 (.21)	17.77 (< .01)
	***Drd2***	7.23 (.02)	2.83 (.13)	4.44 (.06)
	***Drd3***	1.23 (.29)	0.45 (.52)	3.44 (.10)
	***Drd4***	1.61 (.24)	1.28 (.29)	10.47 (.01)
**NAc**	***Maoa***	24.24 (< .01)	19.94 (< .01)	4.17 (.07)
	***Sert***	0.02 (.89)	0.02 (.89)	0.43 (.53)
	***Tph1***	1.41 (.27)	0.02 (.88)	3.37 (.10)
	***Tph2***	103.77 (< .01)	55.75 (< .01)	22.77 (< .01)
	***Comt***	0.56 (.48)	0.07 (.79)	0.53 (.49)
	***Drd2***	1.49 (.26)	0.09 (.77)	4.61 (.06)
	***Drd3***	N/A		
**PFC**	***Drd4***	11.25 (.01)	0.11 (.75)	0.02 (.91)
	***Maoa***	14.21 (< .01)	0.10 (.76)	0.65 (.44)
	***Sert***	2.10 (.19)	1.65 (.23)	5.63 (.04)
	***Tph1***	0.88 (.38)	0.01 (.96)	0.28 (.61)
	***Tph2***	13.95 (< .01)	0.10 (.78)	1.00 (.34)

Experiment #2. Impulsivity, mTBI, and the Frontostriatal Circuit

#### Hyperactivity

**Fig 5:**
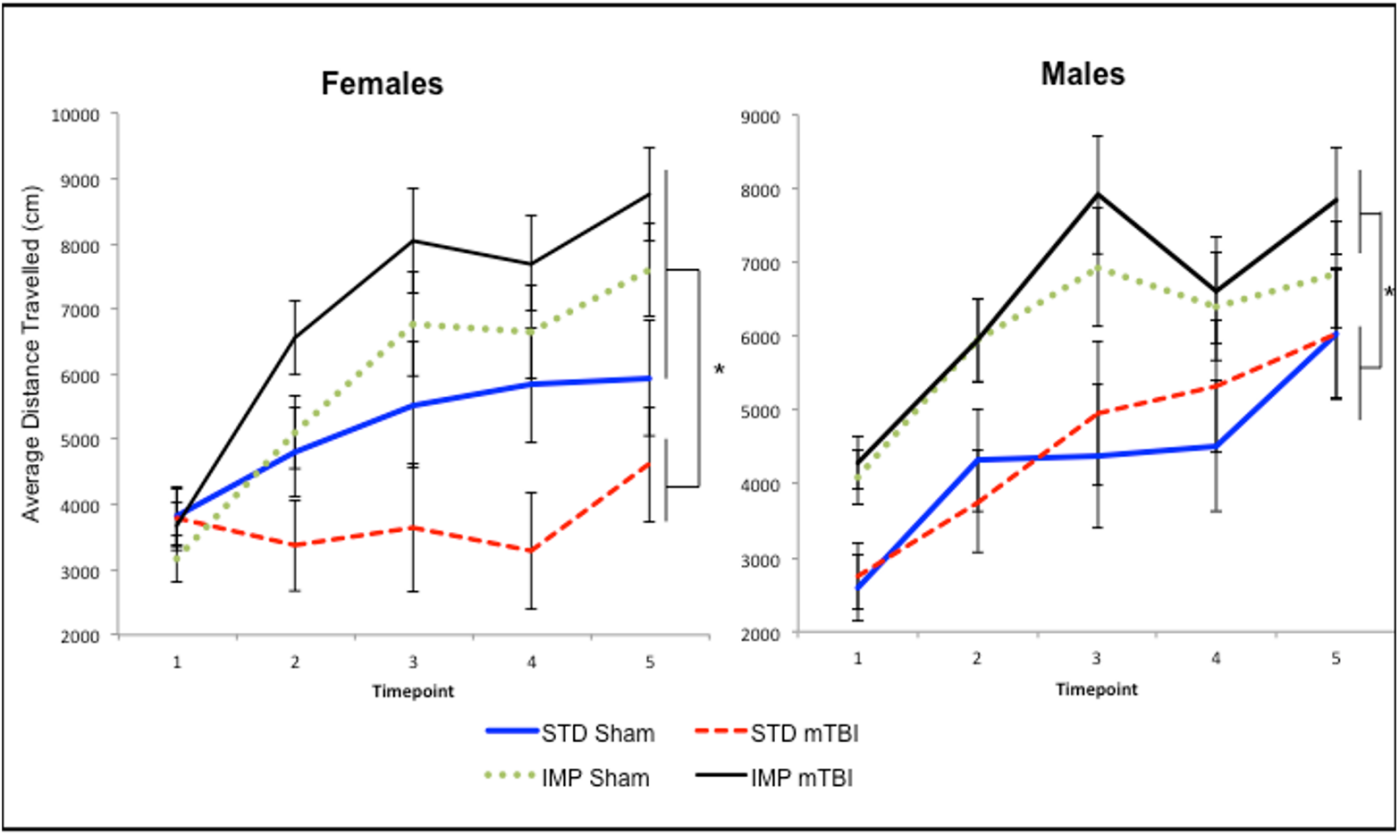
Graphical representation of the average activity level for A) Males, and B) Females, demonstrating that the IMP cohorts were significantly more active than the STD animals at each of the 5 distinct time-periods with STD-mTBI females also showing significant reductions in activity when compared to STD-shams (*main effect of cohort, *p* < .05).

Across all time-points measured, IMP animals were significantly more active in the open field than STD animals, both before and after administration of the mTBI. The repeated measures ANOVA, demonstrated a significant test day x sex x cohort effect, *F*(4, 36) = 3.46, *p* = .01. In addition, the test of between subjects effects found a significant main effect of cohort, *F*(1, 41) = 21.88, *p* < .01. See Fig 5.

#### Go/No-Go Paradigm

**Fig 6:**
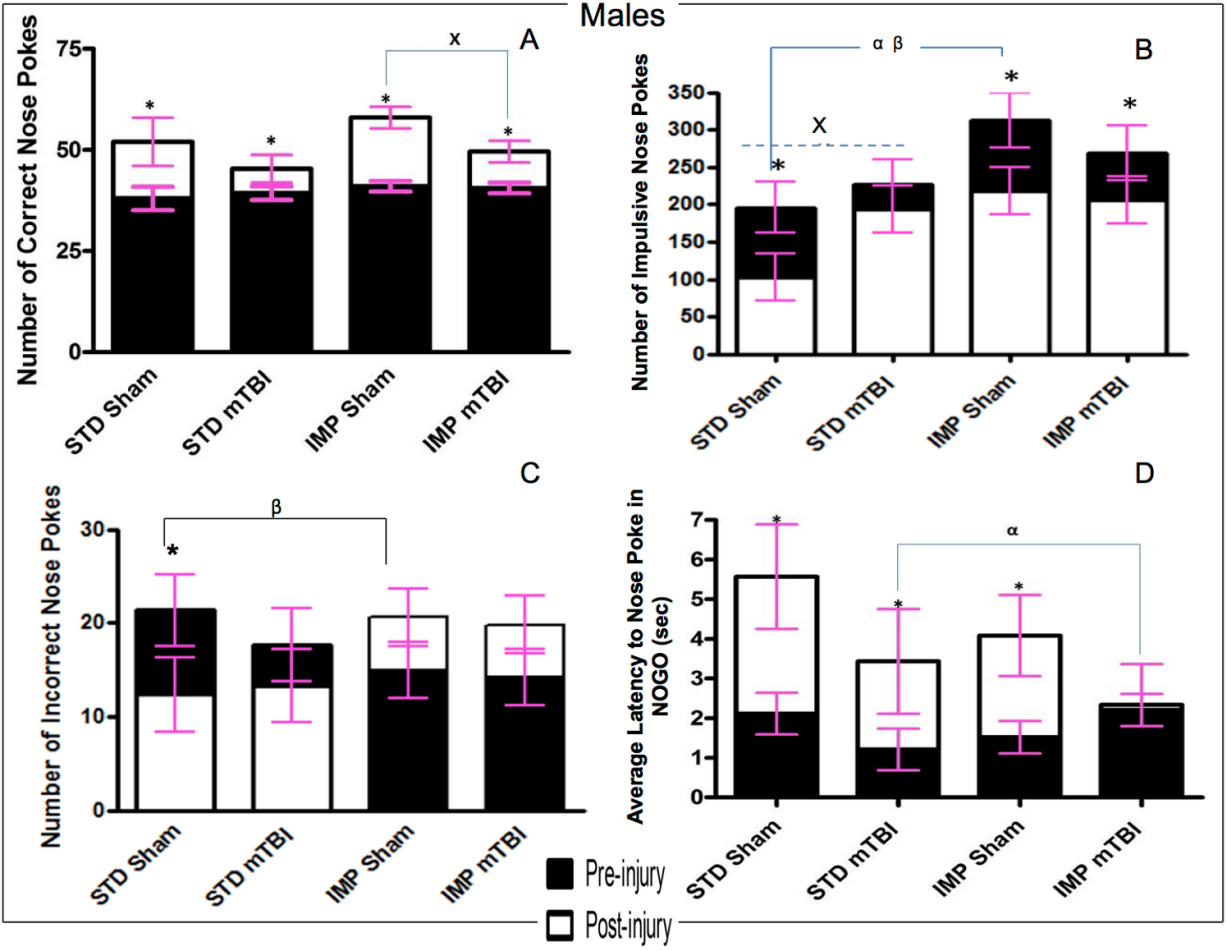
Graphical representation of Go/No-Go results. A) accuracy, B) Impulsivity, C) Inaccuracy, and D) Response Inhibition, for **male** animals from the STD and IMP cohorts at the pre-injury (black bars) and post-injury (white bars) test sessions. Overall accuracy increased across all cohorts from the pre-injury session to the post-injury session, but the magnitude of change was greater for sham animals. Males in the STD cohort exhibited a reduction in incorrect nose pokes with increased training but animals in the IMP cohort actually exhibited an increase in incorrect nose pokes. Cohort and mTBI increased impulsivity when compared to STD-sham animals. Finally, all males except IMP-mTBIs demonstrated improvements in latency to nose poke on the NOGO trials, but again the magnitude was greatest for STD-shams (α significant effect of IMP at pre-injury, β significant effect of IMP at post-injury, ^x^ significant effect of mTBI, * significant effect of testing period, all *p* < .05).

**Fig 7:**
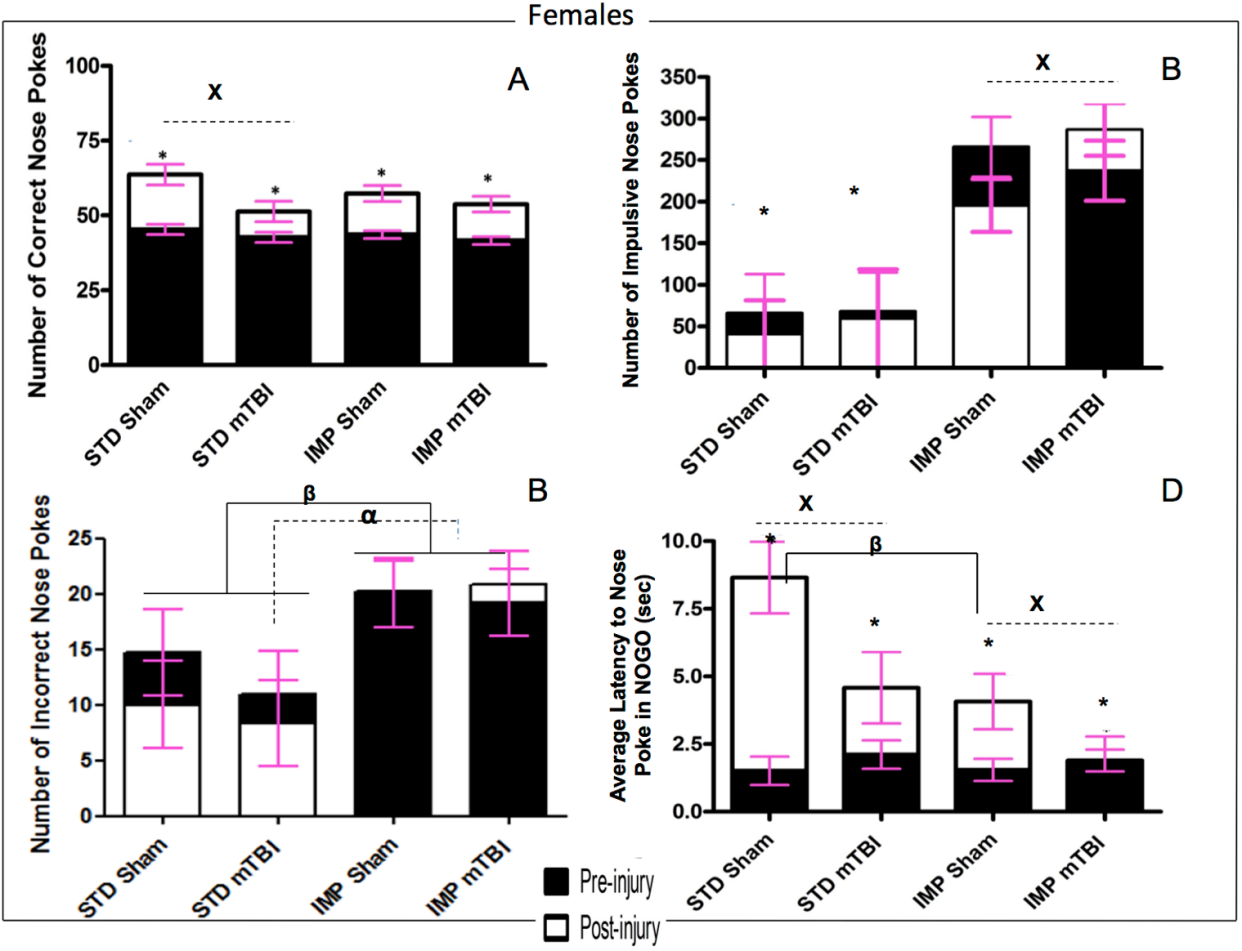
Graphical representation of Go/No-Go results. A) Accuracy, B) Impulsivity, C) Inaccuracy, and D) Response Inhibition, for **female** animals from the STD and IMP cohorts at the pre-injury (black bars) and post-injury (white bars) test sessions. Overall accuracy increased across all cohorts from the pre-injury session to the post-injury session, but the magnitude of change was greater for sham animals. Similar to males, the STD cohort exhibited a reduction in incorrect nose pokes with increased training but animals in the IMP cohort did not exhibit a change in the number of incorrect nose pokes. STD females exhibited significantly fewer impulsive nose pokes than IMP females at both time points. Finally, also like males, all animals except IMP-mTBIs demonstrated improvements in latency to nose poke on the NOGO trials, but the magnitude was greatest for the STD-shams (α significant effect of IMP at pre-injury, β significant effect of IMP at post-injury, x significant effect of mTBI, * significant effect of testing period, all *p* < .05).

Results from the Go/No-Go paradigm are split between Fig 6 (Males) and Fig 7 (Females).

Accuracy: The repeated measures ANOVA found a significant test day x injury effect, *F*(1, 36) = 12.21, *p* < .01; that although all animals improved their accuracy from the original test day to the 2^nd^ testing session, the improvement was much greater for sham animals than mTBI animals across both cohorts and sexes. All animals performed similarly during the first test session, but animals with a sham injury nose-poked significantly more accurately than animals that had experienced a mTBI in the second session. Figs 6A and 7A.

Impulsivity: The number of ‘impulsive’ nose-pokes (nose-poking prior to activation of the Go stimulus, or during a timeout) was measured for both testing sessions. At the first test session, there were no significant differences in impulsivity were found between sham and mTBI animals. However, significant differences were seen for the IMP and STD cohorts, whereby the IMP animals were significantly more impulsive than the STD animals. In addition, for all groups except IMP males, impulsivity increased in the 2^nd^ session for animals with a mTBI, but not for those with a sham injury. See Figs 6B and 7B. The repeated measures ANOVA found a significant test day x sex x cohort effect, *F*(1,36) = 2.59, *p* = .02, and a significant test day x injury effect, *F*(1, 36) = 2.85, *p* = .02.

Inaccuracy: Inaccuracy was a measure of the number of holes that were poked incorrectly while the cued light was on. While animals in the STD cohort exhibited significant improvement on the task from the first test session to the 2^nd^, male animals in the IMP cohort actually performed worse at the second session, whereas IMP females remained static across time points. There was no effect of injury on inaccuracy scores. The repeated measures ANOVA found a significant test day x cohort effect, *F*(1, 36) = 8.44, *p* < .01. In addition, the between subjects test found a significant main effect of cohort, *F*(1, 41) = 6.71, *p* = .01, and a significant sex by cohort interaction, *F*(1, 41) = 3.88, *p* = .05. See Figs 6C and 7C.

Response Inhibition: Animals were scored on their ability to inhibit a response during the No-Go stimuli. Early in the training procedure (test day 1), all animals scored poorly on this task, regardless of cohort or sex. Similar to the accuracy, sham animals exhibited greater improvements from the pre-injury test to the post injury session than mTBI animals. The time-dependent improvements in response inhibition were also greater for animals in the STD cohort than the IMP cohort. The repeated measures ANOVA (sphericity assumed) demonstrated a significant test day x cohort effect, *F*(1, 36) = 9.81. *p* < .01, and a significant test day x injury effect, *F*(1, 36) = 11.42, *p* < .01. See Figs 6D and 7D.

### Extinction Paradigm

#### Accuracy and Missed Lights

**Fig 8:**
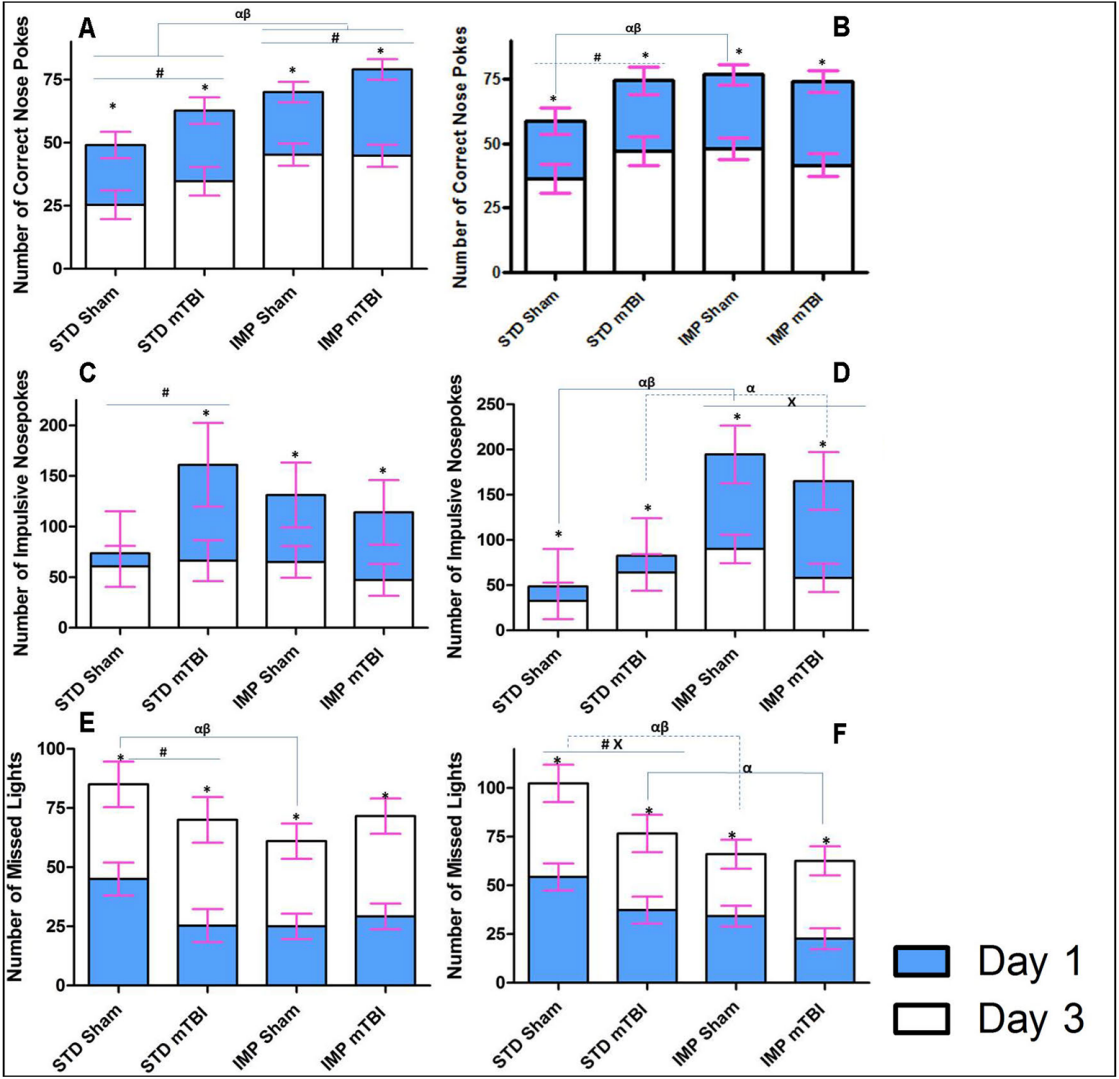
Illustrative representation of the results from the extinction portion of the behavioural testing for STD and IMP animals; blue bars represent the initial testing day and white bars represent the scores after the 3^rd^ consecutive day of no reward in the 5 choice serial reaction task. In this task, rats are no longer rewarded for completing the task as previously trained; therefore learning would entail switching strategies and reducing the number of correct nose pokes. The upper panels (A & B) demonstrate the number of correct lights with STD-shams learning the task quicker; the middle panels (C & D) show that the average number of impulsive nose-pokes (premature or inappropriate) decreased when the reward was removed; and finally (E & F) represent the number of lights that the rats chose to ignore with STD-shams again outperforming all other groups (α significant effect of IMP at Day 1, β significant effect of IMP at Day 3, x significant effect of mTBI, * significant effect of testing period, all *p* < .05).

The accuracy and missed lights measures were used in the extinction paradigm to determine how motivated the rats were to continue the task properly despite the absence of a reward. This would provide a measure of task-switching ability and the degree of habituation that the rats developed to the paradigm. On both accuracy and the number of missed lights, male and female STD shams performed significantly better than all other groups. See Fig 8A and 8C. When focused on male animals, results demonstrated that STD shams were quicker to learn that their behaviour was no longer being rewarded; as they had less accurate lights and more missed lights than all other male animals at the first time point. Although animals in the other groups (STD-mTBI, IMP-Sham, IMP-mTBI) improved over the testing period, STD-Sham animals still missed significantly more lights on the final day of extinction testing. Examination of the females also showed that STD-Shams missed significantly more lights and performed better on the accuracy test at both time points when compared to all other groups. The only exception to this was accuracy levels for STD-mTBI animals at the 2^nd^ test period. The repeated measures ANOVA for accuracy demonstrated significant effects for cohort, sex, injury, cohort x sex, and cohort x injury (*p’s* = .00, .02, .02. .04, and .02, respectively) in the between-subjects analysis. The repeated measures ANOVA for missed lights, demonstrated main effects of cohort (*p* < .01), injury (*p* = .02), and a cohort x injury interaction (*p* = .02) in the between-subjects analysis.

#### Impulsivity

Impulsivity was also measured during the extinction trials to determine if rats were more or less impulsive when the reward was removed from the task. Similar to the accuracy and missed lights measure, STD-Sham animals (both male and female) were significantly less impulsive than animals from all other groups on the first day of testing. No significant differences in impulsivity were seen between male IMP-mTBIs and STD-mTBIs, but differences were significant between IMP-mTBIs and STD-mTBIs females. Females from the IMP cohort were twice as impulsive as the STD cohort on the first extinction trial. On the last day of extinction testing, no differences in impulsivity were found for males; surprisingly, all animals had low levels of impulsivity. In contrast, female animals exhibited an alternating pattern of impulsivity reduction whereby IMP-Sham > STD-mTBI > IMP-mTBI > STD-Sham on the last day of extinction testing. See Fig 8E and 8F. The repeated measures ANOVA found a significant day x cohort interaction, *F*(1, 36) = 5.68, *p* = .03, and a significant day x sex x cohort interaction, *F(*1, 36) = 3.91, *p =* .05. The between-subjects test revealed a significant effect of cohort (*p* = .05), and a cohort x sex interaction (*p* = .05).

### Molecular Analysis

**Fig 9:**
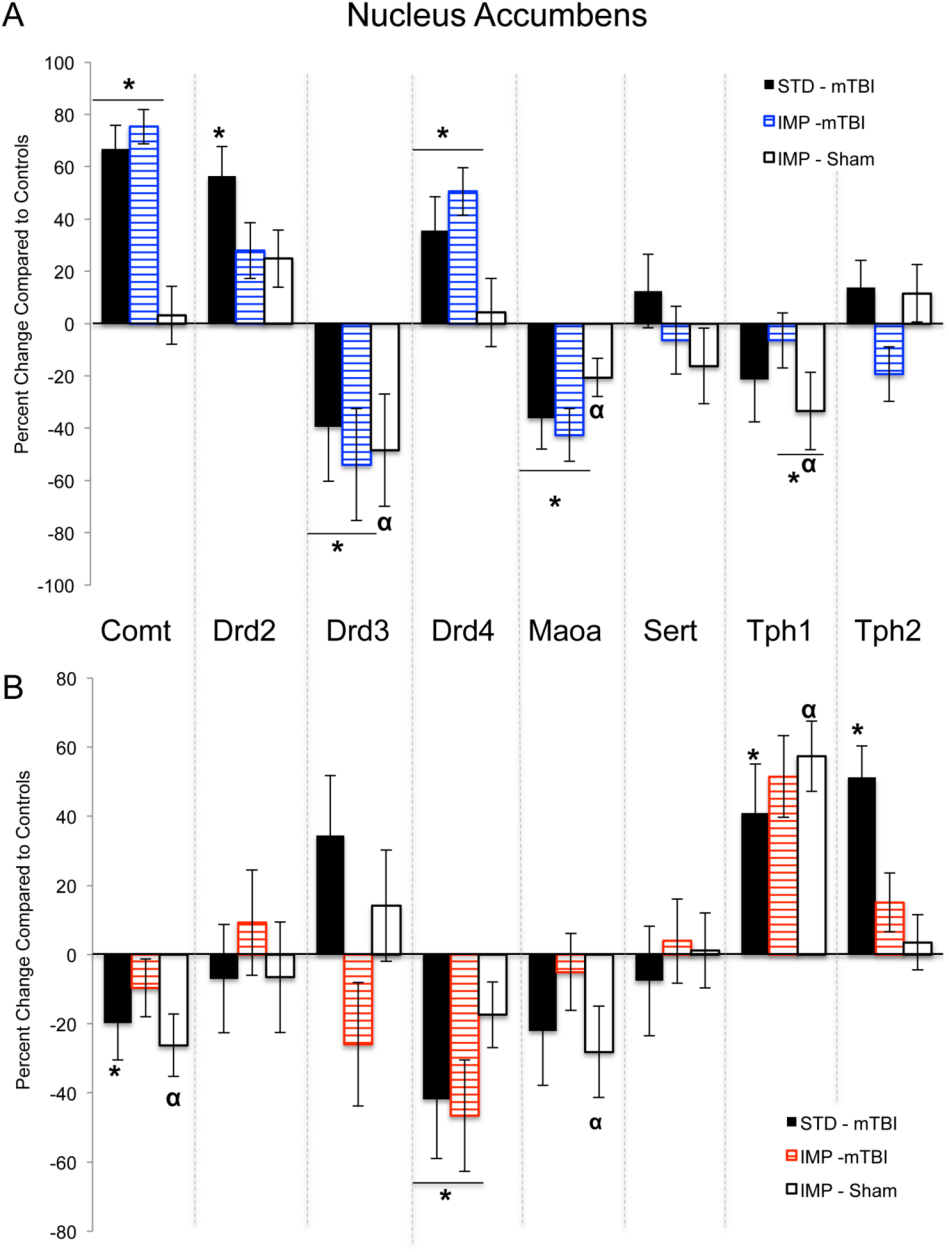
Changes in PFC gene expression for STD animals with a mTBI and IMP animals from both groups. Males are in the upper panel (A) and females in the lower panel (B). The centerline of each graph represents typical expression (STD-shams) with the bars exhibiting percent changes from normal (^*^effect of mTBI, ^α^ effect of cohort *p* < .05).

**Fig 10:**
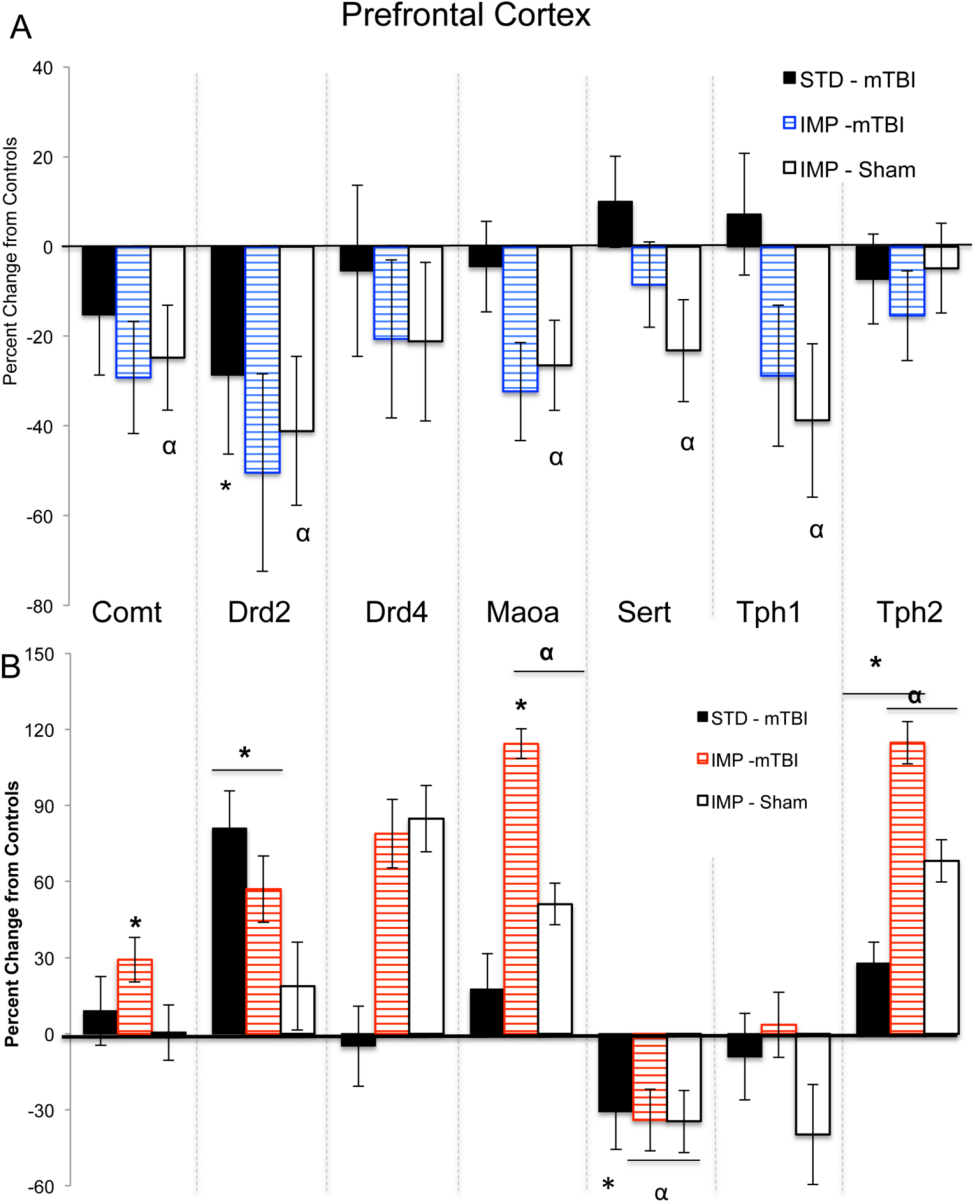
Changes in NAc gene expression for STD animals with a mTBI and IMP animals from both groups. Males are in the upper panel (A) and females in the lower panel (B). The centerline of each graph represents typical expression (STD-shams) with the bars exhibiting percent changes from normal (^*^effect of mTBI, ^α^ effect of cohort, *p* < .05).

Results from the three-way ANOVAs for changes in gene expression can be found in Table 3. Because 8 distinct genes (*Comt*, *Drd2*, *Drd3*, *Drd4*, *Maoa*, *Sert*, *Tph1*, and *Tph2*) were analyzed in two brain regions, the data are represented in table format to simplify presentation and comprehension. Briefly, in the PFC significant main effects of sex were demonstrated in the majority of the genes, whereby males and females would display significant but opposing changes in expression when comparing standard shams to impulsive shams and in response to the mTBI. Sex differences were also present in the NAc with males and females displaying opposing changes in gene expression. In the NAc changes in *Drd4* expression were the best predictor of a mTBI for both sexes; but in the PFC of females, *Drd4* changes were indicative of the IMP. Changes in *Maoa* and *Tph1* expression in the PFC were the best predictors of impulsivity for males. Illustrative demonstration of the epigenetic modifications can be found in Fig 9 for the PFC and Fig 10 for the NAc.

**Table 3:** Summary of Three-Way ANOVA results for genes examined in the nucleus accumbens and prefrontal cortex of impulsive and standard rats with and without a mTBI (F (*p*), bold text denotes significant effects).

	Gene	Sex Effect	Injury Effect	Cohort Effect	Sex x Injury	Sex x Cohort	Injury x Cohort	3-Way Interaction
	***Comt***	1.35 (.25)	**12.39 (< .01)**	0.18 (.68)	**14.44 (< .01)**	1.05 (.32)	3.19 (.09)	2.28 (.15)
	***Drd2***	**10.72 (< .01)**	2.25 (.15)	0.01 (.91)	1.29 (.27)	0.08 (.78)	0.52 (.47)	2.71 (.11)
	***Drd3***	0.92 (.35)	2.12 (.16)	**7.03 (.01)**	1.62 (.22)	1.31 (.26)	0.04 (.85)	**5.02 (.03)**
NAc	***Drd4***	0.37 (.55)	0.94 (.34)	0.20 (.66)	**21.19 (< .01)**	1.67 (.21)	0.53 (.47)	0.06 (.80)
	***Maoa***	**21.87 (< .01)**	**9.03 (< .01)**	3.09 (.09)	**9.39 (< .01)**	1.10 (.30)	**4.79 (.04)**	0.47 (.50)
	***Sert***	0.94 (.34)	0.18 (.67)	0.28 (.60)	0.47 (.50)	1.45 (.24)	0.05 (.84)	0.11 (.74)
	***Tph1***	0.00 (.99)	0.70 (.41)	0.50 (.49)	0.23 (.64)	3.41 (.07)	0.35 (.56)	**5.59 (.02)**
	***Tph2***	**44.72 (< .01)**	**4.71 (.04)**	**4.60 (.04)**	**10.50 (< .01)**	0.44 (.51)	**10.3 (< .01)**	0.04 (.84)
	***Comt***	0.09 (.77)	0.10 (.75)	0.61 (.44)	2.88 (.10)	3.54 (.07)	0.80 (.38)	0.03 (.87)
	***Drd2***	0.25 (.62)	0.33 (.57)	**4.10 (.05)**	**8.58 (< .01)**	3.50 (.07)	0.00 (.98)	1.52 (.23)
	***Drd3***	N/A						
PFC	***Drd4***	0.43 (.52)	0.06 (.81)	1.78 (.20)	0.01 (.98)	**8.56 (< .01)**	0.01 (.91)	0.02 (.88)
	***Maoa***	0.06 (.80)	2.36 (.14)	2.08 (.16)	**5.72 (.03)**	**35.2 (< .01)**	1.13 (.29)	1.38 (.25)
	***Sert***	3.34 (.08)	0.08 (.78)	**8.46 (< .01)**	**4.06 (.05)**	0.01 (.98)	1.87 (.18)	1.06 (.32)
	***Tph1***	0.03 (.87)	1.76 (.19)	**7.27 (.01)**	0.17 (.69)	1.76 (.19)	1.96 (.18)	1.57 (.22)
	***Tph2***	**6.25 (.01)**	0.54 (.47)	**5.94 (.02)**	**4.30 (.05)**	**11.7 (< .01)**	0.05 (.82)	0.23 (.63)

## Discussion

As demonstrated in previous study that was conducted our laboratory [31], exposure to a mTBI/concussion during the juvenile period was associated with increased impulsivity, deficits in spatial attention, and reduced response inhibition. Building upon our prior study, the current experiment demonstrated that the mTBI-induced behavioural changes were linked to alterations in spiny neuron morphology (increased dendritic arbourization and reduced spine density) and gene expression changes in the PFC and NAc. The cohort of animals that were impulsive prior to the injury exhibited a differential pattern of change in the dopamine- and serotonin-related genes, when compared to controls and mTBI animals, in addition to an exacerbation of mTBI-induced deficits in response inhibition and cognitive flexibility.

### Pediatric mTBI and the Frontostriatal Pathway

Impairment in executive functioning is often reported in children after an early brain injury, with specific complaints regarding inhibitory control and impulsivity [40, 41]. Using the 5-choice serial reaction task and the Go/No-Go paradigm, the current study demonstrated that similar to humans, animals exposed to a single mTBI, exhibited decreased spatial attention, increased impulsivity, and impaired response inhibition. Male mTBI animals were significantly more likely than sham animals to exhibit impulsive behaviours, whereas both male and female animals with an early injury demonstrated deficits in spatial attention and a reduced ability to correctly nose poke the illuminated hole. The brain injury also induced neuroanatomical changes in the medium spiny neurons of the NAc. The NAc is a key structure involved in the cortical circuits required for top-down frontostriatal mediation of reward and inhibition [7]. A previous study conducted in our laboratory found that the single mTBI also altered dendritic morphology and spine density of pyramidal neurons in the mPFC [42]. Changes to the structure of neurons in these two brain regions would alter the functionality and developmental trajectory of the reward and attentional neural circuits. Although a causal relationship was not established, it is possible that the reductions in NAc spine density may have been associated with the mTBI-induced impulsivity. Spontaneously hypertensive rats that are often used to model inattention and impulsivity in the lab also exhibit reduced NAc spine density [43] and rats bred for impulsive traits display reductions in DA receptors localized to NAc neurons [12], also suggesting a reduction in spine density. Interestingly, our study also demonstrated that the NAc neurons exhibited significant increases in dendritic arbourization, which may be a compensatory mechanism to accommodate the reduction in spine density. Stimulant exposure increases both dendritic branching and spine density in the NAc [44] suggesting that stimulant-induced impulsivity is functionally different from the impulsive behaviours associated with mTBI. In addition,

The comprehensive examination of the dopaminergic and serotonergic genes of the frontostriatal pathway predicted to be involved in impulsivity following mTBI clearly illustrate significant sex and region dependent differences. In both male and female mTBI animals, significantly more genes were altered in the NAc (5/8 and 6/8 respectively) when compared to those altered in the PFC (2/8 and 3/8). More specifically, expression of the dopamine receptors, *Drd2*, *Drd3*, and *Drd4* were significantly affected by mTBI. Prior studies in male rats examining distinct forms of impulsivity identified similar reductions in PFC *Drd2* mRNA [45]. However, we identified mTBI-induced reductions in NAc *Drd3*, but increases in *Drd2*, whereas other studies of impulsivity have noted decreases in both for male animals [12, 45]. These differences however, may reflect distinctions between inherent impulsivity and induced impulsivity. Conversely, the female mTBI animals in this study exhibited opposing changes, characterized by increased *Drd2* in the PFC, increased *Drd3* in the NAc and reduced *Drd4* in the NAc. To our knowledge no other studies have examined impulsivity-related changes in gene expression for females, making it difficult to draw comparisons, but sex-differences in epigenetic responses are not uncommon [46].

The serotonergic connections between the NAc and PFC appear to play a larger role in the response to mTBI for females than for males. Females exhibited significant enhancement of *Tph1/2* (genes involved in the synthesis of 5HT) in the NAc that was not present in males, in addition to modifications in their *Sert* and *Tph2* expression levels in the PFC. Experimental manipulations in rats that reduce serotonin function have been shown to impair response inhibition [5], using the task in this study that exhibited the largest mTBI-effect in females. Studies have indicated that serotonin involvement is likely critical for certain aspects of impulsivity and executive function [5]. Finally, expression of the two genes involved in metabolism of dopamine, serotonin, and norepinephrine (*Comt* and *Maoa*) were modified in the NAc but unaltered in the PFC. Similar to the DA receptors, changes in expression of *Comt* were opposite for males and females, whereas *Maoa* was only reduced in males. Given a strong association between catecholamine degradation and the bioavailability of their receptors, the changes in receptor levels and metabolic enzymes reflect systematic dysfunction of critical feedback loops. The sex-dependent changes in *Comt* and the serotonergic pathways may contribute to differences in symptom presentation and treatment response. For example, methylphenidate, which acts upon *Comt* mediated dopamine transmission, is the most commonly prescribed drug for ADHD but is ineffective for a large portion of children, with some studies finding differential efficacy rates based upon the sex of the individual [47, 48]. Greater understanding of symptom etiology and sex differences may aid in the development of more efficacious therapeutic strategies.

### Impulsivity, mTBI, and the Frontostriatal Circuit

The second aspect of this study sought to determine if inherent impulsivity would affect frontostriatial-related behavioural and molecular outcomes following an early brain injury. Epidemiological research indicates that impulsive children are at greater risk for brain injuries and concussion because they engage in risky behaviours and are less concerned about consequences [49]. In addition, children with ADHD seem to fare worse after a concussion, suffering from more significant and long-lasting inattentive and hyperactive symptomology, combined with prolonged return to baseline functioning (see [28, 50] for a review of the literature). The current study demonstrated that the presence of impulsive and hyperactive behaviour prior to the injury exacerbated some of the mTBI-induced deficits on the Go/No-Go paradigm with effects being more pronounced in females. For females, inherent impulsivity increased the number of improper and premature (impulsive) nose pokes, in addition to exacerbating response inhibition deficits. While impulsive male animals also exhibited worsening of response-inhibition deficits, the changes were not as substantial. The differences in baseline abilities for males and females may be responsible for the distinct patterns identified in the IMP group; standard male animals were less proficient on the No-Go stimuli and much more impulsive at baseline (sham and mTBI) than standard females. The latter findings are consistent with human literature exemplifying much higher baseline rates (10:1) of impulse control problems and ADHD in boys as compared to girls [51].

When examining results from the extinction training, both IMP and mTBI animals demonstrated impairments in cognitive flexibility, as they were less able to stop performing the task when no longer being rewarded; they maintained high levels of correct nose pokes. A study by Belin et al, [52] also found that highly impulsive rats were unable to switch strategies even when they were being punished rather than rewarded. This result may reflect alterations to the reward system that are believed to contribute to ADHD phenotypes. Studies demonstrate that ADHD children tend to habituate more quickly and intensely to rewarding stimuli, but exhibit reduced physiological responses to extinction stimuli [53]. This impaired activation to the presentation of extinction stimuli may help explain an interesting finding in our study, namely that improper and premature responding (impulsive nose pokes) decreased in all groups when the reward had been removed for consecutive days. This is counter-intuitive, as one would expect increased impulsivity when the rules of the paradigm switch without warning. Discontinuation of reinforcing rewards however generally starts an extinction process that is associated with decreased tonic dopamine activity [54]. As impulsivity is believed to be, at least in part attributable to reductions in basal levels of dopamine, further reductions associated with extinction could cause a ‘floor’ effect [54]. The additive effects of these two processes may have been beneficial for this aspect of the test as depletion of dopamine from specific neural circuits such as the striatum and PFC has been shown to improve attentional control [55].

The examination of the serotonergic and dopaminergic genes in the IMP cohort demonstrated that even prior to a mTBI, animals in this group displayed significant dysregulation of the frontostriatal pathway. Serotonin appears to play a greater role in this model of inherent impulsivity; both male and female animals in the IMP cohort displayed some alteration to the expression levels of *Sert*, *Tph1*, and *Tph2* in both the NAc and PFC. The modifications in expression of these genes suggest reduced 5-HT levels, which has been associated with increased impulsivity and premature responding on the Go/No-Go paradigm [56]. The only DA receptor altered in IMP females was the *Drd4* in the NAc, with IMP males exhibiting reductions in *Drd2* (NAc) and *Drd3* (PFC). *Maoa* was reduced in the NAc of both males and females, but displayed opposing alterations in the PFC (up in females, down in males). This is interesting, not only because *Maoa* inhibitors have sex-dependent rates of efficacy for neuropsychological impairments, but also because the same *Maoa* haplotypes have been demonstrated to confer protection or risk for ADHD depending on the sex of the individual [57].

When combining the mTBI with the IMP phenotype, we found an exacerbation of *Comt*, *Maoa*, and *Tph2* in the PFC of females, such that the changes in expression were additive and produced a substantial modification that was above the change associated with mTBI or IMP alone. This epigenetic response is consistent with the behavioural changes, whereby females displayed a significant worsening of behaviours when the two phenotypes were combined. Males did not exhibit this intensification of behavioural deficit, nor an additive epigenetic response. When examining gene expression, IMP-mTBI males exhibit similar changes in NAc expression to that of mTBI animals, whereas in the PFC, IMP-mTBI males display changes that closely mimic those of the IMP animals. IMP-mTBI males therefore exhibited significant but differential dysregulation of the two primary regions in the frontostriatal circuit, whereas females exhibited intensification of the PFC related deficits. Although males and females differed with respect to the underlying pathophysiology, both patterns of dysfunction could explain why impulsive/ADHD individuals fare worse after a mTBI [50].

## Conclusion

Our results demonstrate that early mTBI/concussions can induce a secondary-ADHD like phenotype characterized by impulsivity and inattention. This behavioural phenotype is associated with modifications to the structure of medium spiny neurons in the NAc, as well as changes in expression of dopaminergic and serotonergic genes critical to the function and communicational abilities of the frontostriatal circuit. In addition, we found that inherent impulsivity exacerbated behavioural and epigenetic alterations associated with early mTBI/concussions, but these outcomes were sex-dependent and related to differential pathogenic processes. Importantly the study has shown that despite similar behavioural phenotypes, the underlying causes of impulsivity can vary significantly. In addition, the sex differences and etiological differences in the serotonergic and dopaminergic modifications exemplify the need to tailor treatment strategies to the specifications of the premorbid characteristics of the individual.

## Supporting Information

S1 File Data for each of the rats with respect to performance on the 5-Choice Serial Reaction task for the testing dates and changes in gene expression at the time of sacrifice. (XLS)

S2 File Data for dendritic branch order, dendritic length and spine density based upon Golgi-Analysis of brain tissue collected at the time of sacrifice. (XLS)
